# Synthesis of [^13^C_4_]-labeled ∆^9^-Tetrahydrocannabinol and 11-*nor*-9-Carboxy-∆^9^-tetrahydrocannabinol as Internal Standards for Reducing Ion Suppressing/Alteration Effects in LC/MS-MS Quantification

**DOI:** 10.3390/molecules190913526

**Published:** 2014-09-01

**Authors:** Morten Karlsen, Huiling Liu, Jon Eigill Johansen, Bård Helge Hoff

**Affiliations:** 1 Department of Chemistry, Norwegian University of Science and Technology, Høgskoleringen 5, 7491 Trondheim, Norway; 2 Chiron AS, Stiklestadveien 1, 7041 Trondheim 7041, Norway; E-Mails: Karlsen@chiron.no (M.K.); Huiling.Liu@chiron.no (H.L.); Huiling.Liu@chiron.no (J.E.J.)

**Keywords:** ^13^C-labeled standards, [^13^C_4_]-olivetol, [^13^C_4_]-∆^9^-tetrahydrocannabinol, [^13^C_4_]-11-*nor*-9-carboxy-∆^9^-tetrahydrocannabinol

## Abstract

(−)-∆9-Tetrahydrocannabinol is the principal psychoactive component of the cannabis plant and also the active ingredient in some prescribed drugs. To detect and control misuse and monitor administration in clinical settings, reference samples of the native drugs and their metabolites are needed. The accuracy of liquid chromatography/mass spectrometric quantification of drugs in biological samples depends among others on ion suppressing/alteration effects. Especially, ^13^C-labeled drug analogues are useful for minimzing such interferences. Thus, to provide internal standards for more accurate quantification and for identification purpose, synthesis of [^13^C_4_]-∆^9^-tetrahydro-cannabinol and [^13^C_4_]-11-*nor*-9-carboxy-∆^9^-tetrahydrocannabinol was developed via [^13^C_4_]-olivetol. Starting from [^13^C_4_]-olivetol the synthesis of [^13^C_4_]-11-*nor*-9-carboxy-∆^9^-tetrahydrocannabinol was shortened from three to two steps by employing nitromethane as a co-solvent in condensation with (+)-apoverbenone.

## 1. Introduction

(−)-∆^9^-Tetrahydrocannabinol (∆^9^-THC, **1**), is a product of the female flowering parts of *Cannabis sativa* (marijuana), and is the main psychoactive substance in the plant. As compound **1** and analogues act on the cannabinoid receptors, the cannabinoid group of compounds is also medicinally useful. Among others, development have led to the ∆^9^-THC-containing drug Sativex^®^, indicated for treatment of moderate to severe spasticity due to multiple sclerosis [1], and Marinol^®^, used in the treatment of chemotherapy-induced nausea and vomiting [2]. The ∆^9^-THC analogue Nabilone™, also an approved drug, is indicated efficient in treatment of the same conditions [2,3]. However, recreational use and continued illicit use of marijuana have increased the importance of having methods to determine usage by individuals.

**Scheme 1:**
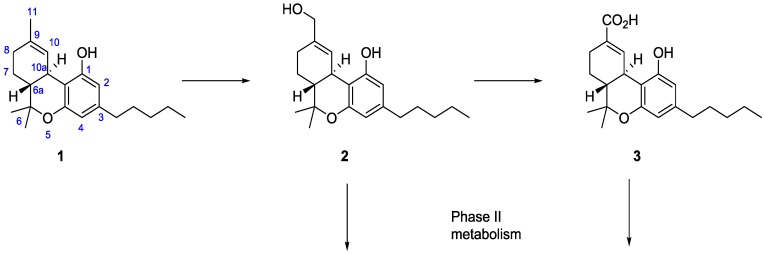
Major phase I metabolites derived from ∆^9^-THC (**1**).

*In vivo* ∆^9^-THC (**1**) undergoes phase I metabolism to yield 11-hydroxy-∆^9^-tetrahydrocannabinol (11-OH-THC, **2**) peaking immediately after smoking, Scheme 1 . Metabolite **2** is also psychoactive, but is rapidly oxidized to the inactive metabolite (‒)-11-*nor*-9-carboxy-∆^9^-tetrahydrocannabinol (THC-COOH, **3**), which slowly increases and plateaus after 2–4 h [4]. Metabolism occurs mainly in the liver by cytochrome P450 enzymes 2C9, 2C19, and 3A4 [5]. The main urinary metabolites occur as phase II conjugates of glucuronic acid, and less commonly as sulphate, glutathione, amino acids, and fatty acid conjugates. The main site for glucuronidation is the carboxylate at C-11, but 11-OH-THC (**2**) may undergo phase II metabolism as well.

Efficient analysis of both drugs and their metabolites are required in the fields of pharmacology, clinical toxicology and forensic toxicology, but also for workplace drug testing, testing of driving under the influence of drugs, doping analysis and rehabilitation programs. Since these analyses are often performed by mass spectroscopic techniques, reference samples of the native drug, its main metabolites and suitable standards are needed for identification and quantification purposes. Stable isotope labeled internal standards (SIL IS) are added to correct for error in the sample preparation and co-eluting substances that could alter or suppress the signal [6]. Deuterated internal standards with varying degree of labeling are currently used as IS in quantification of cannabinoids [7,8,9,10,11].

However, under certain conditions the MS ionization of the analytes is influenced differently from that of its deuterium labeled analogues, due to slight differences in retention. This potentially leads to inaccuracies in the quantification [12,13]. Urine analysis of ∆^9^-THC (**1**) and its metabolites by LC-MS/MS is especially challenging, due to the presence of unstable compounds and strong adsorption to hydrophobic surfaces and the need for enzymatic pre-processing of the sample prior to analysis. Further, the importance of an effective sample clean-up prior to cannabinoid analysis to remove matrix interferences and maintaining a high extraction efficiency has been highlighted [14]. Also, Scheidweiler *et al*. [15] concluded that urine samples from different individuals gave matrix effects not observed during method validation, contributing to inaccurate cannabinoid quantification. It is also likely that the challenge with ion suppressing/alteration or matrix effects will be even more pronounced by the use of high resolution techniques such as UHPLC-MS/MS. This indicates that there is a need for a better SIL IS for cannabinoid analysis. ^13^C-Labeled IS are particularly suitable for minimizing ion suppression/alteration effects in LC-MS/MS analysis, therefore the quantitative analysis in various biological samples are particularly accurate and reproducible. However, to avoid “overlap” with the natural ^13^C in the native compound in MS detection, the number of labeled atoms must preferably be at least three. Recently, the success of substituting deuterated IS with ^13^C IS has been shown in the case of amphetamine and methamphetamine quantification [16]. The use of ^13^C as labeled compounds as IS is also widely known and appreciated in other fields of analytical chemistry [17,18,19,20,21]. Based on this background we disclose herein a synthesis of [^13^C_4_]-labeled Δ^9^-THC (**1**) and Δ^9^-THC-COOH (**3**) made via [^13^C_4_]-olivetol.

## 2. Results and Discussion

### 2.1. Strategy Selection and Synthesis of [^13^C_4_]-Olivetol

**Scheme 2:**
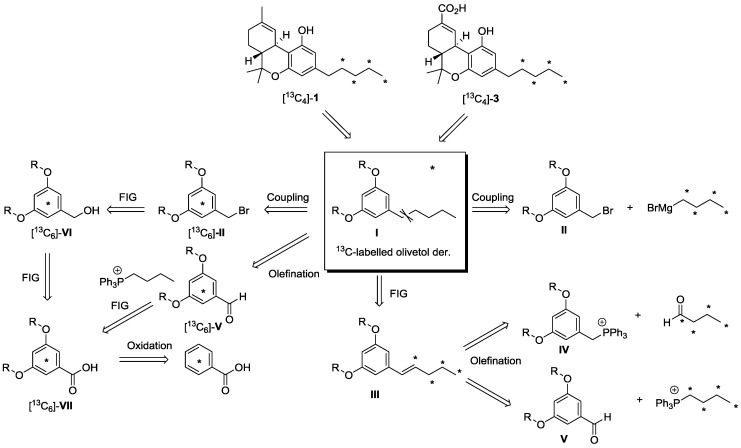
Retrosynthetic route to ^13^C-labeled olivetol derivatives.

Our aim was to find an efficient synthesis of ^13^C-labeled (−)-(6a*R*,10a*R*)-6,6,9-trimethyl-3-pentyl-6a,7,8,10a-tetrahydro-6H-benzo[*c*]chromen-1-ol, termed Δ^9^-THC (**1**), and its main urine metabolite Δ^9^-THC-COOH (**3**). Previously developed routes towards Δ^9^-THC (**1**) involve both reactions with 5-pentyl-1,3-benzenediol (olivetol) and various chiral natural derived terpenes [22,23,24,25] and asymmetric syntheses [26,27,28]. Herein, we have focused on the use of terpenes for introducing the correct stereochemistry. Due to the lengthy and rather complicated synthesis of the terpenes, it was decided to label olivetol. Moreover, labeled olivetol (**I**) is also a useful building block for the second target, namely Δ^9^-THC-COOH (**3**). The goal was to introduce at least three [^13^C] atoms in olivetol, and several strategies were considered, see Scheme 2.

**Scheme 3:**
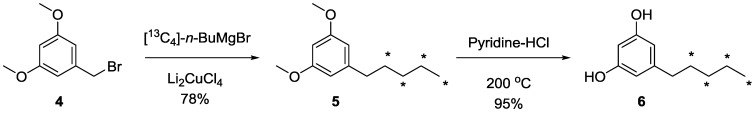
Synthesis of [^13^C_4_]-olivetol (**6**).

A key intermediate was assumed to be protected 5-(bromomethyl)benzene-1,3-diol derivatives, **II**, which can be converted to olivetol analogues (**I**) by metal catalysed coupling [29] or olefination chemistry [30] using **IV** or **V**. Another precursor is [^13^C_6_]-3,5-dihydroxybenzoic acid (**VII**), available by synthesis from [^13^C_6_]-benzoic acid [31]. Depending on the following strategy (olefination or C-C coupling) additional steps are needed. As the benzoic acid routes appeared long with expected lower ^13^C atom efficiency, we decided to prepare an olivetol derivative with four carbons in the alkyl chain by using commercially available [^13^C_4_]-*n*-bromobutane. The chemistry performed is shown in Scheme 3

A Wurtz type reaction between 1-(bromomethyl)-3,5-dimethoxy-benzene (**4**) and the Grignard reagent made from [^13^C_4_]-*n*-bromobutane in the presence of dilithium tetrachlorocuprate gave 78% isolated yield of [^13^C_4_]-1-(3,5-dimethoxyphenyl)pentane (**5**). The major by-product observed was the 3,5-dimethoxybenzyl dimer. By-product formation was somewhat increased by running the reaction in tetrahydrofuran instead of diethyl ether. To obtain [^13^C_4_]-olivetol (**6**) the dimethoxy ether groups were removed by heating compound **5** in pyridine hydrochloride at 200 °C. Trimethylsilyl iodide could also be used, although purification was more tedious in this case. The Wittig strategy using butyltriphenylphosphonium salt in combination with the non-labeled benzaldehyde (**V**) was also briefly tested. The first step involving formation of the Wittig salt was slow in toluene and needed several days at reflux to reach full conversion. On the other hand both the alkene forming step, and the hydrogenation of the olefin with palladium on carbon as catalyst proceeded smoothly. Thus, by tuning of the first step, this route might be highly useful.

### 2.2. [^13^C_4_]-Labeled Δ^9^-THC and Δ^9^-THC-COOH

**Scheme 4:**
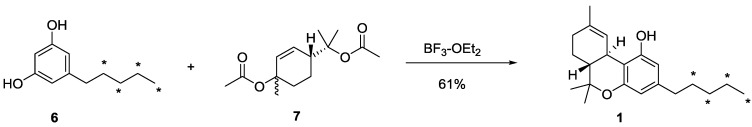
Synthesis of [^13^C_4_]-Δ^9^-THC (**1**).

In the synthesis of [^13^C_4_]-Δ^9^-THC (**1**), [^13^C_4_]-olivetol (**6**) was simply condensed with the terpene **7** in the presence of boron trifluoride as Lewis acid following the procedure of Silverberg *et al*. [25], Scheme 4. Alternative acid catalysts for such transformations has been published by Rosati *et al*. [32]. After purification by preparative HPLC the product was isolated in 61% yield as a colourless oil (purity >99%). The product gradually darkened upon storage at −20 °C, however, no change in chromatographic purity was noticed.

**Figure 1:**
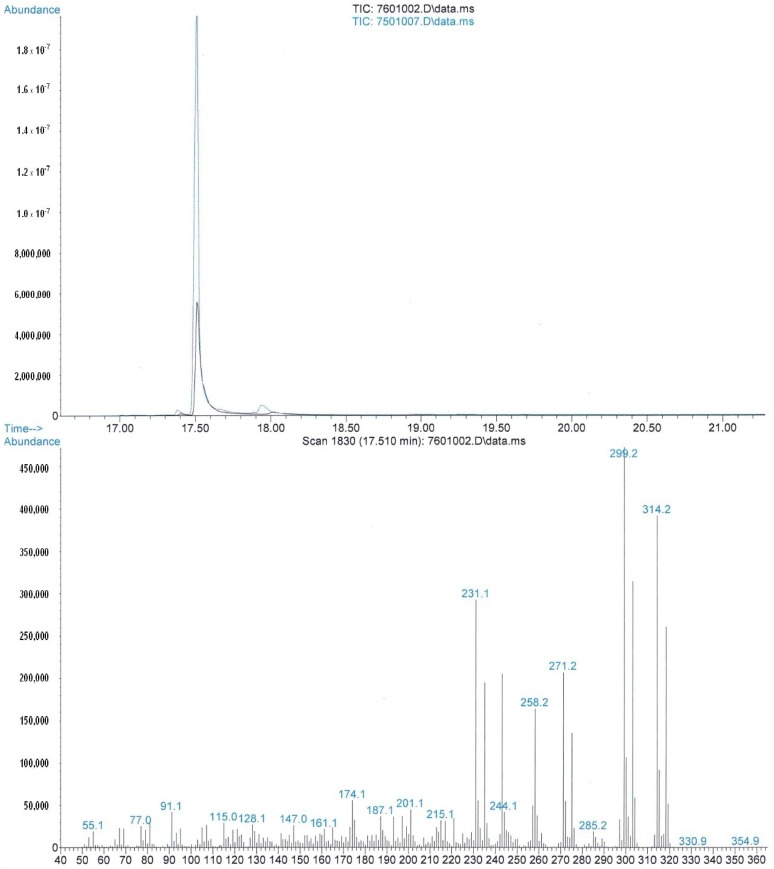
Comparison of GC elution and the total ion chromatograms of [^13^C_4_]-Δ^9^-THC (**1**) and native Δ^9^-THC.

[^13^C_4_]-Δ^9^-THC (**1**) was analysed by GC-MS and the retention times were compared with that of native Δ^9^-THC obtained by hexane extraction of a *Cannabis Sativa* hybrid strain. The superimposed chromatograms showing identical elution, and the overlaid total ion chromatograms of [^13^C_4_]-Δ^9^-THC (**1**) and native Δ^9^-THC are shown in Figure 1.

**Scheme 5:**
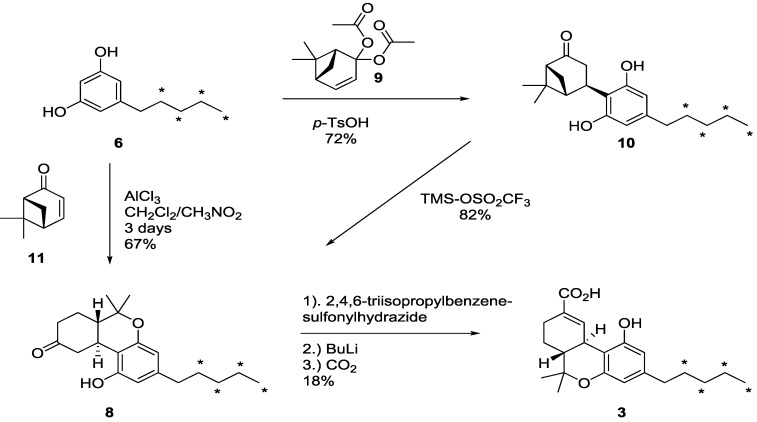
Synthesis of [^13^C_4_]-Δ^9^-THC-COOH (**3**) from [^13^C_4_]-olivetol (**6**).

Synthesis of [^13^C_4_]-Δ^9^-THC-COOH (**3**), was more complex than that of **1**. Based on previous reported total synthesis, we decided to utilise ketone **8** as a late stage precursor (Scheme 5).

Ketone **8** was obtained in two steps from (+)-nopinone diacetate (**9**), and [^13^C_4_]-olivetol (**6**) [33]. First, an acid catalysed stereoselective and regioselective Michael type addition gave [^13^C_4_]-**10**. Compared to the previous report the yield was increased from 33% to 72% by using two equivalents of the terpene **9** [33]. Treating [^13^C_4_]-**10** with trimethylsilyl triflate gave [^13^C_4_]-**8** in 82% isolated yield.

An alternative procedure towards **8** that was previously used in the synthesis of the structurally related compound Nabilone™ [34] was also investigated. By this one-step reaction between (1*R*,5*R*)-6,6-dimethylbicyclo[3.1.1]hept-3-en-2-one ((+)-apoverbenone, **11**) and 5-(1,1-dimethylheptyl)resorcinol in the presence of aluminum chloride, a low 16% yield was reported [34]. However, we were able to increase the yield in this transformation up to 67% by the use of nitromethane as co-solvent in the condensation between [^13^C_4_]-olivetol (**6**) and **11**. Although the exact role of nitromethane has not been investigated it is assumed that the improvement in part is due to increased solubility and mixing of aluminum chloride. Nitromethane has also been reported to modify/reduce the activity of the aluminum chloride, which might also be an important aspect [35]. It should be noted that both of the terpenes **9** and **11** in the end yielded the natural enantiomeric form of (-)-Δ^9^-THC-COOH (**3**) in contrast to other possible strategies [36].

**Figure 2:**
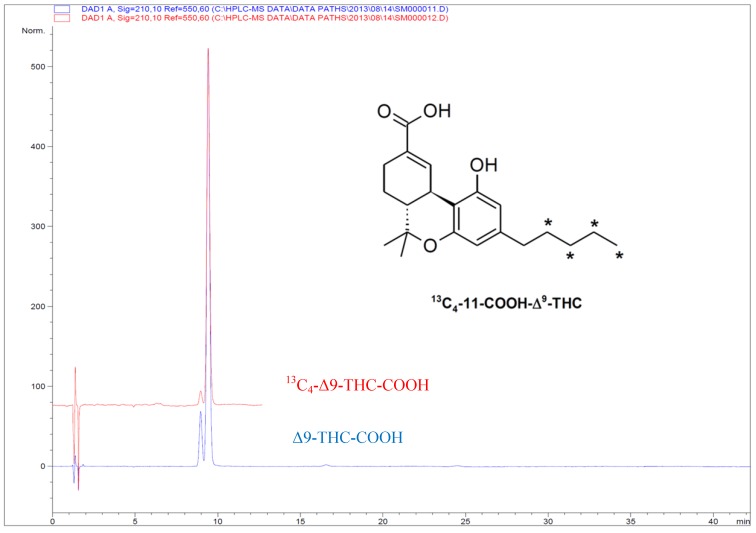
LC chromatogram of [^13^C_4_]-Δ^9^-THC-COOH (**3**) as compared to native Δ^9^-THC-COOH. The impurities are [^13^C_4_]-Δ^8^-THC-COOH and Δ^8^-THC-COOH, respectively.

The precursor **8** was then transformed to [^13^C_4_]-Δ^9^-THC-COOH (**3**) in three operations. Treatment with triisopropylbenzenehydrazine yielded the corresponding hydrazone, which was carefully dried prior to treatment with butyl lithium in *n*-hexane in the presence of tetramethylethylenediamine (TMEDA) and then gaseous CO_2_. Scrupulously dry and pure reagents were needed in this transformation. Kachensky *et al*. [37] reported on a 9/1 ratio of Δ^9^/Δ^8^ THC-COOH by using a 10% solution of TMEDA. The dilution factor was however not mentioned. Following these conditions, only the Δ^8^-isomer was obtained in our earlier test reactions. This is in line with that observed by Nikas *et al.* [33]. Decreasing the concentration by fifty percent by addition of more of the TMEDA/*n*-hexane solvent mixture had a positive effect. The crude product contained a 6/4 ratio of [^13^C_4_]-**3** and the Δ^8^-regioisomers, which corresponds with that reported by Nikas *et al*. [32]. Purification was done first by silica-gel column chromatography to remove structurally unrelated impurities, followed by two crystallisations to arrive at a final purity of 97.5%. A chromatogram of the prepared material as compared to the native substance is shown in Figure 2. Preparative HPLC was used to recover additional [^13^C_4_]-Δ^9^-THC-COOH (**3**) from the mother liquor giving a total yield of 18%. The enantiomeric excess of Δ^9^-THC-COOH (**3**) was determined by self-induced non-equivalence by ^1^H-NMR spectroscopy of ketone intermediate [^13^C_4_]-**8** [38] and was found to be 96% ee. 

## 3. Experimental Section

### 3.1. Chemicals and Analysis

Bulk solvents were purchased either from LabScan (Gliwice, Poland) or Merck (Darmstadt, Germany). Deuterated solvents were purchased from CDN Isotopes Inc (Pointe-Claire, QC, Canada). All chemicals or reagents used were of highest purity available and purchased from Sigma-Aldrich (Oslo, Norway) or Acros (Geel, Belgium). All solvents and chemicals were used as is without further purification unless otherwise stated. Anhydrous solvents were used as is and stored over activated molecular sieves. The silica-gel used for flash chromatography was Merck silica gel 60 (230–400 mesh). For chromatography, thin layer chromatography (TLC) silica gel 60F_254_ Merck plates/sheets were employed with visualization under UV light at 254 nm. ^1^H and ^13^C-NMR spectra were recorded from Bruker Advance DPX instruments (400/100 MHz). Chemical shifts (δ) are reported in ppm rel. to tetramethylsilane. Due to the high intensity of the ^13^C-labeled carbons as compared to those unlabeled, and multiple coupling, some NMR resonances were not detected. In this study the non-labeled benzoic carbonyl signal at around 166 ppm was difficult to detect, experiencing extensive coupling by the neighbouring ^13^C-isotopes. Isotopic purity and accurate mass determination in positive and negative mode on the final product was performed on a “Synapt G2-S” Q-TOF instrument from Waters with a resolution of 5 ppm. Samples were ionized by the use of an atmospheric pressure solids analysis probe (ASAP). No chromatography separation was used prior to the mass analysis. HPLC analysis was performed on an Agilent 1200 with atmospheric pressure chemical ionization (APCI)/ electrospray ionization (ESI) multimode ionization. Acetonitrile was used as the mobile phase in combination with water buffered at pH 3 with formic acid. Column used was XBridge™ C18, 5 µm, 4.6 × 150 mm from Waters or Hilic Plus, 3.5 µm, 4.6 × 100 mm from Agilent. GC-MS analysis was performed on Agilent 6890 with EI ionization, Agilent column HP5MS, 30 m, 0.25 mm internal diameter, 0.25 µm film. Preparative HPLC of [^13^C_4_]-∆^9^-THC (**1**) and [^13^C_4_]-∆^9^-THC-COOH (**3**) was performed with 19 × 150 mm XBridge prep C18, 5 μm column purchased from Waters. [^13^C_4_]-∆^9^-THC (**1**): eluent: acetonitrile/water (90/10), flow: 11 mL/min, run time 9 min; [^13^C_4_]-∆^9^-THC-COOH (**3**): acetonitrile /20 mM formic acid in water (60/40) hold 5 min, ramp to 90% acetonitrile, flow: 14 mL/min, run time 15 min. The identity of all products and intermediates were confirmed by co-elution with unlabeled materials and comparison of NMR spectra to confirm their structures.

### 3.2. Synthesis of [^13^C_4_]-1-(3,5-Dimethoxyphenyl)pentane (5)

A solution of [^13^C_4_]-butyl magnesium bromide (3.50 mmol) cooled to 0–5 °C, generated from [^13^C_4_]-butyl bromide and magnesium in ether (3.5 mL, 1 M), and was added dropwise to a mixture of 3,5-dimethoxybenzyl bromide (**14**, 0.71 g, 3.07 mmol) in diethyl ether (20 mL) and dilithium tetrachlorocuprate (0.1 mL, 0.1 M in tetrahydrofuran) also at 0–5 °C. The reaction was allowed to warm slowly to room temperature and then stirred for 8 h. The reaction mixture was quenched by addition of saturated ammonium chloride solution (5 mL) followed by 10% sulphuric acid (25 mL). The organic layer was separated and the aqueous layer was extracted with diethyl ether (20 mL). The resulting emulsion was passed through glass wool and the organic layers were combined and dried over magnesium sulphate. The product was purified by silica-gel column chromatography (*n*-heptane/acetone, 93/7, R_f_ = 0.35) yielding 0.51 g (2.40 mmol, 78%) of pure [^13^C_4_]-3,5-dimethoxyphenyl-1-pentane as a clear liquid; purity: 99% (GC-MS): 212.2 (29), 168.1 (11), 152.1 (100), 137.1 (7), 121.1 (5); ^1^H-NMR (400 MHz, CHCl_3_) δ: 0.74–1.15 (m, 3H), 1.15–1.31 (m, 2H), 1.43–1.62 (m, 3H), 1.73–1.91 (m, 1H), 2.51–2.66 (m, 2H), 3.76–3.88 (m, 6H), 6.35 (t, *J* = 2.3 Hz, 1H), 6.40 (d, *J* = 2.3 Hz, 2H); ^13^C-NMR (101 MHz, CHCl_3_) δ: 14.0 (dd, *J* = 34.4, 3.7 Hz), 22.5 (ddd, *J* = 34.8, 32.1, 2.2 Hz), 30.6–31.9 (m, 2C), 35.8–36.6 (m), 55.1 (2C), 97.5, 106.4 (2C), 145.3, 160.6 (2C).

### 3.3. Synthesis of [^13^C_4_]-olivetol (**6**)

[^13^C_4_]-3,5-Dimethoxyphenyl-1-pentane (**5**, 0.51 g, 2.40 mmol) was mixed with pyridinium hydrochloride salt (8.00 g) under argon. The mixture was heated at 200 °C until no more starting material was detected by GC (2 h). The reaction mixture was cooled to 22 °C, water added (20 mL), and the reaction mixture extracted with toluene (3 × 20 mL). The organic phase was dried over magnesium sulphate, and then evaporated in *vacuum*. The product was purified by silica-gel column chromatography (toluene/diethyl ether, 5/1, R_f_ = 0.42) yielding 0.42 g (2.28 mmol, 95%) of [^13^C_4_]-olivetol (**6**) as a semisolid colourless liquid that crystallized upon standing to light pink crystals; purity: 99.5% (GC/MS): 184.1 (32), 138.1 (9), 124.1 (100); ^1^H-NMR (400 MHz, DMSO-*d*_6_) δ: 0.65–1.04 (m, 3H), 1.05–1.20 (m, 2H), 1.26–1.51 (m, 3H), 1.56–1.73 (m, 1H), 2.36 (tt, *J* = 7.5, 3.9 Hz, 2H), 5.91–6.10 (m, 3H), 8.99 (br. s., 2H); ^13^C-NMR (101 MHz, DMSO-*d*_6_) δ: 13.9 (dd, *J* = 34.4, 3.7 Hz), 22.0 (ddd, *J* = 34.8, 31.1, 2.2 Hz), 30.0–31.4 (m, 2C), 34.9–35.5 (m), 99.9, 106.3 (d, *J* = 1.5 Hz, 2C), 143.9–144.4 (m), 158.1 (2C).

### 3.4. Synthesis of [^13^C_4_]-(−)-∆^9^-THC (**1**)

A 25 mL round bottom flask was dried in the oven, fitted with a septum and cooled. The flask was thoroughly flushed with argon. (+)-*p*-Menth-2-ene-1,8-diacetate (**7**, 0.46 g, 1.82 mmol) and [^13^C_4_]-olivetol (**6**) (0.34 g, 1.85 mmol) were added. Anhydrous dichloromethane (15 mL) was added and stirred under an argon atmosphere. The solution was cooled at −5 °C and boron trifluoride diethyl etherate (243 µL, 1 eq) was added. The solution gradually darkened to red. After 20 min, the reaction was quenched with 10% Na_2_CO_3_ (5 mL). The layers were separated and the organic layer was washed with 10% Na_2_CO_3_ (20 mL). The combined aqueous phases were extracted once with dichloromethane (20 mL). The organic solutions were combined and washed with water (20 mL) and brine (20 mL), and then dried over magnesium sulphate. The crude yield was 1.32 g of a brown oil, which was purified further by preparative HPLC to yield 0.36 g (1.13 mmol, 61%) of [^13^C_4_]-(−)-∆9-THC (**1**); purity ≥ 99%; (GC-MS) 318.2 (84), 303.2 (100), 275.2 (42), 258.2 (20), 243.1 (23), 235.1 (60), 221.1 (7), 197.1 (9); ^1^H-NMR (400 MHz, CDCl_3_) δ: 0.88 (dm, *J* = 124.2 Hz, 3H), 1.10 (s, 3H), 1.08–1.53 (m, 7H), 1.42 (s, 3H), 1.66–1.77 (m, 1H), 1.69 (s, 3H) 1.89–1.96 (m, 1H), 2.14–2.21 (m, 2H), 2.44 (td, *J* = 7.6, 1.6 Hz, 2H), 3.21 (dm, *J* = 11.0 Hz, 1H), 4.76 (s, 1H), 6.15 (d, *J* = 1.5 Hz, 1H), 6.28 (d, *J* = 1.6 Hz, 1H), 6.30–6.34 (m, 1H); ^13^C-NMR (101 MHz, CDCl_3_) δ: 14.0 (dd, *J* = 34.6, 3.7 Hz), 19.3, 22.5 (t, *J* = 33.9 Hz), 23.4, 25.0, 27.6, 30.6 (dd, *J* = 33.9, 3.7 Hz), 31.2, 31.5 (t, *J* = 33.9 Hz), 33.6, 35.3–35.7 (m), 45.8, 77.1–77.2 (m), 107.5 (d, *J* = 1.5 Hz), 109.0, 110.1 (d, *J* = 1.6 Hz), 123.7, 134.4, 142.8 (dd, *J* = 3.7, 1.6 Hz), 154.1, 154.8; HRMS (ES^+^): calcd for ^13^C_4_C_17_H_31_O_2_ [M+H]^+^: 319.2458; found 319.2461.

### 3.5. Synthesis of [^13^C_4_]-(4R)-4-(4-pentyl-2,6-dihydroxy-[^13^C_6_]-phenyl)-6,6-di-methyl-2-norpinanone (**10**)

*p*-Toluenesulphonic acid monohydrate (1.60 g, 8.37 mmol) was added to a degassed solution of [^13^C_4_]-olivetol (**6**, 1.10 g, 5.98 mmol) and (+)-6,6-dimethyl-2,2-diacetoxy-3-norpinene (**9**, crude mixture, 2.80 g, 11.7 mmol) in chloroform (100 mL) at 0 °C under an argon atmosphere. The reaction mixture was warmed to room temperature and stirred for 3 days. Water was added (2 mL) and the reaction further stirred for 30 min. The mixture was diluted with diethyl ether (30 mL) and washed sequentially with water (20 mL), saturated aqueous sodium bicarbonate (20 mL), and brine (20 mL). The organic phase was dried over magnesium sulphate and the solvent was removed under reduced pressure. The residue obtained was dry flashed on silica-gel (*n*-heptane/diethyl ether, 55/45) in a Büchner funnel to remove unreacted terpenes. Further purification by silica-gel flash column chromatography, (*n*-heptane/acetone, 7/3, R_f_ = 0.35) gave 1.38 g, (4.33 mmol, 72%) of [^13^C_4_]-**10** as a yellow semisolid. GC-MS: 320.2 (60), 304.2 (27), 277.1 (51), 260.1 (10), 267.1 (18), 237.1 (100), 222.1 (24), 197.1 (63), 150.0 (48), 123.0 (14), 83.0 (36); ^1^H-NMR (400 MHz, CDCl_3_) δ: 0.89 (t, *J* = 6.8 Hz, 3H), 1.00 (s, 3H), 1.16–1.34 (m, 4H), 1.37 (s, 3H), 1.47–1.65 (m, 2H), 2.32 (t, *J* = 5.2 Hz, 1H), 2.39–2.46 (m, 2H), 2.46–2.57 (m, 2H) 2.61 (t, *J* = 5.1 Hz, 1H), 2.66 (dd, *J* = 18.8, 8.7 Hz, 1H), 3.50 (dd, *J* = 19.0, 7.6 Hz, 1H), 3.96 (t, *J* = 8.2 Hz, 1H), 5.36 (br. s., 2 H), 6.19 (s, 2H); ^13^C-NMR (101 MHz, CDCl_3_) δ: 14.02 (dd, *J* = 34.4, 3.7 Hz), 22.2, 22.5 (dd, *J* = 35.1, 33.7 Hz), 24.5, 26.2, 29.5, 30.4–32.1 (m, 2C), 35.2, 38.0, 42.2, 46.8, 57.96, 108.8 (2C), 113.7, 142.7, 155.0 (2C), 217.8.

### 3.6. Synthesis of [^13^C_4_]-(6aR,10aR)-6,6a,7,8,10,10a-Hexahydro-1-hydroxy-6,6-dimethyl-3-pentyl-9H-dibenzo[b,d]pyran-9-one (**8**)

Trimethylsilyl trifluoromethanesulphonate (3.81 mL, 0.3 M solution in nitromethane, 1.14 mmol) was added to a solution of (4*R*)-4-(4-[^13^C_4_]-pentyl-2,6-dihydroxyphenyl)-6,6-dimethyl-2-norpinanone (10, 961 mg, 3.02 mmol) in anhydrous dichloromethane/nitromethane (3/1, 90 mL) at 0 °C under an argon atmosphere. The reaction mixture was stirred for 6 h. The reaction was quenched with saturated aqueous sodium bicarbonate/brine (1/1, 20 mL). Diethyl ether (20 mL) was added. The organic phase was separated, and the aqueous phase was extracted with more diethyl ether (20 mL). The organic phase was washed with brine (20 mL), and dried over magnesium sulphate. Solvent evaporation and purification by silica-gel flash chromatography (toluene/ethyl acetate, 85/15, R_f_ = 0.39) afforded 790 mg (2.47 mmol, 82%) of [^13^C_4_]-**8** as a white foam; purity: 94% (HPLC); (GC-MS): 320.2 (100), 305.1 (34), 277.1 (17), 260.1 (54), 237.1 (84), 197.1 (16), 150.1 (43); ^1^H-NMR (400 MHz, CDCl_3_) δ: 0.69–1.09 (m, 3H), 1.09–1.24 (m, 2H), 1.13 (s, 3H), 1.33–1.61 (m, 4H), 1.48 (s, 3H), 1.65–1.85 (m, 1H), 1.97 (td, *J* = 12.1, 2.8 Hz, 1H), 2.08–2.24 (m, 2H), 2.38–2.56 (m, 3H), 2.59–2.71 (m, 1H), 2.90 (td, *J* = 12.1, 2.8 Hz, 1H), 4.18 (dt, *J* = 15.2, 2.8 Hz, 1H), 6.25 (s, 1H), 6.28 (s, 1H), 7.84 (s, 1H); ^13^C-NMR (101 MHz, CDCl_3_) δ: 14.0 (dd, *J* = 34.4, 3.7 Hz), 18.8, 22.5 (dd, *J* = 35.1, 33.7 Hz), 26.9, 27.8, 30.4–32.1 (m, 2C), 34.9, 35.5 (dd, *J* = 32.9, 4.4 Hz), 40.8, 44.9, 47.4, 76.6, 107.8 (d, *J* = 1.5 Hz), 107.9, 109.0 (d, *J* = 1.5 Hz), 143.5 (dd, *J* = 3.7, 1.5 Hz), 154.5, 155.5, 215.1.

### 3.7. Synthesis of [^13^C_4_]-(6aR,10aR)-6,6a,7,8,10,10a-Hexahydro-1-hydroxy-6,6-dimethyl-3-pentyl-9H-dibenzo[b,d]pyran-9-one (**8**) Using **11**

(+)-Apoverbenone (**11**, 300 mg, 2.20 mmol) was dissolved together with [^13^C_4_]-olivetol (**6**, 0.34 g, 1.85 mmol) in anhydrous dichloromethane/nitromethane (2/1, 5 mL) at 0 °C under an argon atmosphere. To this solution fresh anhydrous aluminium chloride is added in small portions (247 mg, 1.85 mmol). The reaction mixture was stirred for 3.5 days. The reaction was poured on crushed ice and diethyl ether was added. The organic phase was separated, and the aqueous phase was extracted with more diethyl ether (20 mL). The organic phase was washed with brine (20 mL), and dried over magnesium sulphate. Solvent evaporation and purification by silica-gel flash chromatography (toluene/ethyl acetate 85/15, R_f_ = 0.39) afforded 397 mg (1.24 mmol, 67%) of [^13^C_4_]-**11** as a white foam 94% (GC/MS), which can be crystallized from dichloromethane and pentane for higher purity. The spectroscopic properties were identical to that reported in Section 3.6.

### 3.8. Synthesis of [^13^C_4_]-(−)-∆^9^-THC Acid (**3**)

Compound [^13^C_4_]-**8** (138 mg, 0.43 mmol) and 2,4,6-triisopropylbenzenesulphonylhydrazide (128 mg, 0.43 mmol) were mixed in anhydrous toluene (10 mL). After 1 h reaction time at 22 °C, the solvent was evaporated under reduced pressure to give benzenesulphonic acid, 2,4,6-tris(1-methylethyl)-2-[(6a*R*,10a*R*)-6,6a,7,8,10,10a-hexahydro-1-hydroxy-6,6-dimethyl-3-pentyl-9*H*-dibenzo[b,d]pyran-9-ylidene]hydrazide as a foam which were further dried over phosphorous pentoxide and vacuum overnight. This material was dissolved in a mixture of dry *n*-hexane freshly distilled over sodium /TMEDA from a new bottle (6 mL, 1/1 ratio) under an argon atmosphere at −78 °C. *n*-Butyl lithium (387 µL, 0.96 mmol, 2.5 M solution in *n*-hexane) was added to this solution. The reaction mixture was stirred for 20 min at −78 °C and then it was warmed to −5 °C over a 10 min period and stirred at this temperature for an additional 20 min. The reaction mixture was cooled to −78 °C and a second portion of *n*-butyl lithium (194 µL, 0.77 mmol) was added. Following the addition, the mixture was stirred for 10 min at −78 °C and then allowed to warm to 0 °C over a 10 min period. Stirring was continued for 20 min at 0 °C or until N_2_ evolution ceased, and then dry CO_2_ was bubbled into the reaction mixture for 30 min. The pH was adjusted to 2 by the addition of 5% aqueous HCl solution at 0 °C, and the mixture was warmed to room temperature and extracted with diethyl ether (2 × 30 mL). The ethereal solution was washed with brine (20 mL), dried (magnesium sulphate), and the solvent was evaporated under vacuum. The residue obtained was purified by dry flash chromatography on silica gel (*n*-heptane/ethyl acetate, 7/3) to give a mixture of [^13^C_4_]-(−)-∆^9^-THC-COOH (**3**) and [^13^C_4_]-(−)-∆8-THC-COOH (6/4) as a semisolid oil (135 mg, 0.39 mmol). This residue was boiled in *n*-hexane (20 mL) and a few drops of chloroform were added until nearly all the material dissolved. The solution was then slowly cooled to give a gelatinous mixture which was filtered to give 41 mg of a white solid material with a ratio of ∆^9^/∆^8^/ratio of 7/3. This material was further recrystallized from chloroform (0.5 mL) which was slowly diluted with pentane by evaporative diffusion to give the product as white needles (25 mg, 0.07 mmol, 11%) with 97.5% purity [^13^C_4_]-(−)-∆^9^-THC acid and 2.5% of [^13^C_4_]-(−)-∆^8^-THC acid. The mother liquors containing product were further purified by preparative HPLC which also yielded more of the [^13^C_4_]-(−)-∆9-THC-COOH purity ≥ 99%; (total yield 18%); mp 201.9–202.6 °C; ^1^H-NMR (400 MHz, CDCl_3_) δ: 0.66–1.06 (m, 3H), 1.07–1.22 (m, 2H), 1.11 (s, 3H), 1.32–1.53 (m, 4H), 1.43 (s, 3H), 1.71 (t, *J* = 11.1 Hz, 2H), 2.00 (dd, *J* = 12.6, 7.3 Hz, 1H), 2.34–2.49 (m, 3H), 2.55 (dd, *J* = 19.0, 6.3 Hz, 1H), 3.36 (d, *J* = 10.9 Hz, 1H), 6.16 (s, 1H), 6.24 (s, 1H), 8.10 (d, *J* = 1.5 Hz, 1H), -OH and COOH protons could not be detected; ^13^C-NMR (101 MHz, CDCl_3_) δ: 14.0 (dd, *J* = 34.4, 3.7 Hz), 19.0, 22.5 (dd, *J* = 35.1, 33.7 Hz), 24.2, 25.2, 27.5, 29.8–32.3 (m, 2C), 34.6, 35.1–35.9 (m), 44.3, 77.1, 106.8, 107.5, 109.5, 128.5, 143.1 (m), 144.7, 154.6, 154.7, 171.1; HRMS (ES^+^): calcd for ^13^C_4_C_17_H_28_O_4_Na [M+Na]^+^: 371.2019; found 371.2018.

## 4. Conclusions

Synthetic routes towards [^13^C_4_]-∆^9^-THC and [^13^C_4_]-∆^9^-THC-COOH have been developed for their use as stable isotope labeled internal standards. These compounds are especially suited to minimise impact of ion suppressing/alteration effect in LC/MS-MS quantification. To the best of our knowledge preparation of these ^13^C-labeled compounds is being here for the first time. Several methods were tested at each step to identify synthetic routes that were efficient, fast, high- yielding and allowing for a straight forward purification. Both [^13^C_4_]-∆^9^-THC and [^13^C_4_]-∆^9^-THC-COOH were prepared via [^13^C_4_]-olivetol made in-house from 1-(bromomethyl)-3,5-dimethoxybenzene and [^13^C_4_]-*n*-butylmagnesium bromide in the presence of dilithium tetrachlorocuprate. Synthesis of [^13^C_4_]-∆^9^-THC proceeded in 61% yield from [^13^C_4_]-olivetol. In preparation of [^13^C_4_]-∆^9^-THC-COOH, the yield of the precursor [^13^C_4_]-(6a*R*,10a*R*)-6,6a,7,8,10,10a-hexahydro-1-hydroxy-6,6-dimethyl-3-pentyl-9*H*-dibenzo[b,d]pyran-9-one, was improved by applying nitromethane as co-solvent. The final challenging step gave after a tuning of solvent dilution and amount of tetramethylethylenediamine at best a 6/4 mixture of [^13^C_4_]-∆^9^-THC-COOH and the [^13^C_4_]-∆^8^-THC-COOH isomers. [^13^C_4_]-∆9-THC-COOH was isolated in 18% yield after several purification steps.
